# Altered motivation masks appetitive learning potential of obese mice

**DOI:** 10.3389/fnbeh.2014.00377

**Published:** 2014-10-30

**Authors:** Mazen R. Harb, Osborne F. X. Almeida

**Affiliations:** ^1^NeuroAdaptations Group, Max Planck Institute of PsychiatryMunich, Germany; ^2^Neuroscience Domain, Institute of Life and Health Sciences (ICVS), University of MinhoBraga, Portugal; ^3^ICVS/3B's - PT Government Associate LaboratoryBraga/Guimarães, Portugal

**Keywords:** associative learning, pavlovian conditioning, instrumental conditioning, diet-induced obesity, hedonic preference, motivation, body weight regulation

## Abstract

Eating depends strongly on learning processes which, in turn, depend on motivation. Conditioned learning, where individuals associate environmental cues with receipt of a reward, forms an important part of hedonic mechanisms; the latter contribute to the development of human overweight and obesity by driving excessive eating in what may become a vicious cycle. Although mice are commonly used to explore the regulation of human appetite, it is not known whether their conditioned learning of food rewards varies as a function of body mass. To address this, groups of adult male mice of differing body weights were tested two appetitive conditioning paradigms (pavlovian and operant) as well as in food retrieval and hedonic preference tests in an attempt to dissect the respective roles of learning/motivation and energy state in the regulation of feeding behavior. We found that (i) the rate of pavlovian conditioning to an appetitive reward develops as an inverse function of body weight; (ii) higher body weight associates with increased latency to collect food reward; and (iii) mice with lower body weights are more motivated to work for a food reward, as compared to animals with higher body weights. Interestingly, as compared to controls, overweight and obese mice consumed smaller amounts of palatable foods (isocaloric milk or sucrose, in either the presence or absence of their respective maintenance diets: standard, low fat-high carbohydrate or high fat-high carbohydrate). Notably, however, all groups adjusted their consumption of the different food types, such that their body weight-corrected daily intake of calories remained constant. Thus, overeating in mice does not reflect a reward deficiency syndrome and, in contrast to humans, mice regulate their caloric intake according to metabolic status rather than to the hedonic properties of a particular food. Together, these observations demonstrate that excess weight masks the capacity for appetitive learning in the mouse.

## Introduction

Ingestion of foods in excess of actual energy needs leads to overweight and obesity, conditions that raise an individual's risk to develop non-communicable chronic physical and mental diseases (Moussavi et al., 2007; Gunstad et al., 2010; Danaei et al., 2011; Wormser et al., 2011). To help stem the worldwide rise in overweight and obesity (World Health Organization, 2000), it is imperative to further our understanding of eating behavior. Feeding is an innate behavior, involving cognitive (attention, learning and memory, decision-making), sensory (olfactory, visual, taste, somatosensory) and behavioral (motivation) processes that work in an inter-dependent manner (e.g., motivation can be elicited by novel or previously-learnt sensory rewards) (Berthoud, 2011). Importantly, feeding behavior is also governed by peripheral signaling to the brain about energy levels and satiety state (Berthoud, 2011). Thus, the amount of food consumed by an individual is determined by convergence and integration of a multiplicity of neural and peripheral signals and execution commands that are not easy to dissect.

“Hedonic overdrive” has been recently proposed as an explanation for overeating in humans (Cohen, 2008; Berridge and Kringelbach, 2011; van der Plasse et al., 2013). Briefly, the high reward salience of certain foods leads to their consumption even in states of satiety and/or sufficient energy reserves. Responses to hedonic stimuli depend largely on conditioned learning of environmental cues, well-exemplified by the impact of advertising on food choices and intake (Halford et al., 2008; Petrovich et al., 2007; Jones et al., 2010; Powell et al., 2010; Boyland et al., 2011). One important question in the field relates to the mechanisms that drive excessive eating in overweight and obese human subjects, i.e., *Why can overweight and obese individuals not exert sufficient control over their responses to pleasurable foods?* Since excess body weight can reportedly interfere with cognitive performance, is it plausible, for example, that overweight subjects continue to be more susceptible to conditioning stimuli? (Jansen et al., 2003; Rothemund et al., 2007; Cohen, 2008). Another possibility is that overeating in a state of satiation or in the presence of sufficient energy depots is a sign of “reward deficiency syndrome” and reflects dysregulated motivation (Wang et al., 2004; Blum et al., 2006; Stice et al., 2008; Geiger et al., 2009). These possibilities are by no means exhaustive and may include other deficits, including disrupted energy mobilization and energy sensing.

Laboratory rodents are frequently used in research aimed at dissecting the neural and physiological mechanisms that control feeding behavior and body weight (Speakman et al., 2007). Many published studies have demonstrated that obesity compromises memory (Greenwood and Winocur, 1990; Farr et al., 2008; Mielke et al., 2006; Murray et al., 2009; McNeilly et al., 2001; Valladolid-Acebes et al., 2011). Usually, however, the tests use food as the reinforcing stimulus and interpretation of the results do not consider that obese animals have abundant energy supplies and may therefore be less motivated to perform (Peters et al., 2004; Peters and Langemann, 2009; Shin et al., 2011; Kubera et al., 2012). As a result, the idea that obese animals do not perform well because their cognition or reward sensitivity is disturbed may be misleading and, when translated to humans, may stigmatize persons with eating disorders (Puhl and Heuer, 2010).

The experiments reported here were aimed at clarifying the relative roles of cognition, motivation and energetic state in the control of feeding behavior in adult mice that were of normal body weight, overweight and obese; the last two groups of animals were generated by exposing them to energy-rich diets. Our results show that motivation, and therefore learning in an appetitive conditioning task, is inversely proportional to body weight; notably, body weight generally correlates with total fat mass (see Hariri and Thibault, 2010), fat being a primary energy depot. Moreover, our results demonstrate that mice can trade off the hedonic properties of palatable foods (e.g., milk, sucrose) for energy-denser maintenance diets so as to meet their actual energy needs. In this respect, humans and mice may differ remarkably.

## Materials and methods

### Animals

Male mice (C57BL6 strain, Charles River, Sulzfeld, Germany) were used in these experiments. Animals were housed in pairs under standard laboratory conditions with *ad libitum* access to water, unless specifically mentioned. Experimental procedures were compliant with European Union Directive 2010/63/EU and local regulations.

Variable degrees of overweight were induced by maintaining mice on either a standard laboratory (normal) chow (NC; 11.9 kJ/g, 19% crude protein, 4% crude fat, 6% crude fiber), a low-fat, high-carbohydrate diet (LF-HC; 16.1 kJ/g, 10% from fat, 70% from carbohydrate), or a high-fat, high-carbohydrate diet (HF-HC; 19.8 kJ/g, 45% from fat, 35% from carbohydrate). The NC was purchased from Altromin (Lage, Germany, diet 1324 TPF); LF-HC and HF-HC diets were supplied by Brogaarden (Lynge, Denmark, diets D12450B and D12451, respectively, from Charles River Laboratories). Animals received the diets for 12 (pavlovian and operant conditioning experiments and motivation/“wanting” tests; *n* = 50) or 36 (hedonic preference/“liking” tests; *n* = 47) weeks, from 3 months of age onwards.

Behavioral tests (open field, pavlovian and operant conditioning and tests of motivation and preference) were conducted during the daily phase of darkness (lights off: 07:00). Bussey-Saksida automated touchscreen chambers (Horner et al., 2013) were used for the pavlovian and operant conditioning experiments, as described previously (Harb and Almeida, 2014). The reward used as a reinforcer in pavlovian and operant conditioning and motivation test was a liquid food (15 μl of diluted condensed milk, containing 14% sugar). Before any testing commenced, animals underwent 1 week of habituation to the experimental room and experimenter as well as to the liquid food rewards in the test chambers. All animals were subjected to a calorie-restriction schedule to reduce body weights by 10–15% before behavioral testing and calorie restriction continued throughout, unless otherwise stated.

### Open field test

The open field (OF) test was used to measure locomotor activity and explorative behavior; this test was used to ensure that the different diets and induced changes in body weight did not interfere with the animals' motor abilities or, indirectly, with their attention and motivation states. The OF test was conducted before all other behavioral tests. Testing was done in a white light-illuminated (100 lux) Plexiglas arena (OF; white base: 30 × 30 cm; dark gray walls: 30 cm high), in an otherwise dark room. Activity was recorded over 5 min. using a video camera and results were analyzed using ANY-maze software (Stoelting, Wood Dale, IL). The total distance traveled by each mouse was computed. Mice were placed in the OF arena (5 min/session/d) on 2 consecutive days; the first session was used to habituate the animals to the test environment.

### Pavlovian conditioning

Autoshaping was performed in automated touchscreen chambers (Campden Instruments, Loughborough, UK), as described previously (Horner et al., 2013; Harb and Almeida, 2014). Briefly, mice were trained to associate a 10 s flash of white light (presented on the left-hand side of touchscreen to one half of the animals and on the right-hand side to the rest) with the delivery of a liquid food reward (unconditioned stimulus, US) into the food magazine (food tray entries were registered by infrared light beam-breaks). In each test session (30 presentations), mice received 15 light flashes (conditioned stimulus +, CS+) that were followed by reward delivery and 15 light flashes that were not followed by a reward (CS−) (CS+ and CS− were presented at opposite sides of the touchscreen, with the food tray remaining below the center of the touchscreen throughout) in a randomized order [maximum of 2 consecutive presentations of same stimulus, variable interval (VI) schedule of 10–40 s between each stimulus]. Stimuli were not presented until the mouse was centrally located at the rear of the chamber (detected by an infrared light beam) to eliminate chance approaches to the stimuli. This experimental setup has been shown to ensure that mice have identical opportunities to sample the stimuli in each trial (Bussey et al., 1997). Approach to the stimulus was registered as breaking of an infrared light beam placed directly in front of the stimulus; only the first light beam break was recorded. Conditioned responses (CR) were monitored during once daily test sessions until criterion was reached (70% of correct CS+ approach responses per session for at least 3 consecutive days). Animals that failed to complete the daily test session (30 presentations) within 90 min, over the whole period of conditioning, were excluded from the analysis.

As expected, animals developed different conditioned responses (cf. Flagel et al., 2007; Tomie et al., 2012). Mice that reached the criterion of 70% correct responses/session to the CS+ on at least 3 consecutive days were designated as Sign Trackers (ST). Those that made >80% approaches to the food (US) magazine (<20% approaches to the CS+) were categorized as Goal Trackers (GT), and those that made 20–70% approaches to the CS+ (alternated between CS+ and US with approximately equal frequency) were considered to be Intermediate Trackers (IT) (Harb and Almeida, 2014; also see Supplementary Table 1).

### Operant (instrumental) conditioning

A separate batch of animals was used for this set of experiments. Daily sessions comprised of 20 presentations of a light stimulus at the center of the touchscreen. Animals had to “work” for a reward, delivered in a food-tray at the opposite end of the chamber by nose-poking the stimulus; reward delivery was made as soon as the stimulus was touched (Horner et al., 2013). In order to minimize between-trial interference, a VI schedule (10–40 s) was used. Each mouse experienced 1 daily conditioning session that lasted a maximum of 60 min until it reached criterion (completion of 20 trials in <20 min/session on at least 3 consecutive days). The following parameters were recorded and computed for each operant conditioning session: (i) trials completed/session, (ii) time to complete session, (iii) beam breaks/min, and (iv) stimulus touches/min.

### Tests of motivation and hedonic preference

Motivation for food reward retrieval was examined in two ways:

- Motivation (Harb and Almeida, 2014) was evaluated by monitoring reward retrieval latencies and rate of food-tray entries in touchscreen chambers (Horner et al., 2013). Testing was carried out over 2 daily sessions, each of which consisted of 15 presentations of liquid food reward, delivered at a VI 10–40 s, independent of learning strategies, and only after retrieval of the previously-delivered reward.
- Hedonic preference was examined in a batch of mice that had been maintained on NC, LF-LC, or HF-HC diets for 36 weeks, starting at 3 months of age. Mice were presented with two highly-rewarding isocaloric drinking solutions (15% sucrose or milk whose fat content was 5%) in their home-cages; maintenance chow/water being available *ad libitum* throughout, and fluid consumption was measured at 3, 6, and 24 h. In brief, this protocol allowed assessment of the hedonic preference of the liquid diets, independently of the animals' state of satiety or energy needs. In a second step, mice were food-deprived for 48 h and allowed to choose between the milk and sucrose solutions; testing was done in the home-cage but animals did not have access to their normal chow. This design allowed discrimination between hedonic preference *vs.* energy needs by computing actual calories derived from (each of) the liquid foods as a function of the average daily number of calories derived from the maintenance (NC, LF-HC, HF-HC) chow under normal holding conditions.

### Data analysis

Data analyzed using the statistical software package Prism 5.0 (GraphPad, La Jolla, CA). Data were subjected to either One- or Tow-Way ANOVA, followed by Bonferroni post-test comparisons, or by *t*-tests, as appropriate. The minimum level of significance was set *p* ≤ 0.05.

## Results

### Inverse relationship between efficacy of conditioning to food cues and body mass

**Figure 1:**
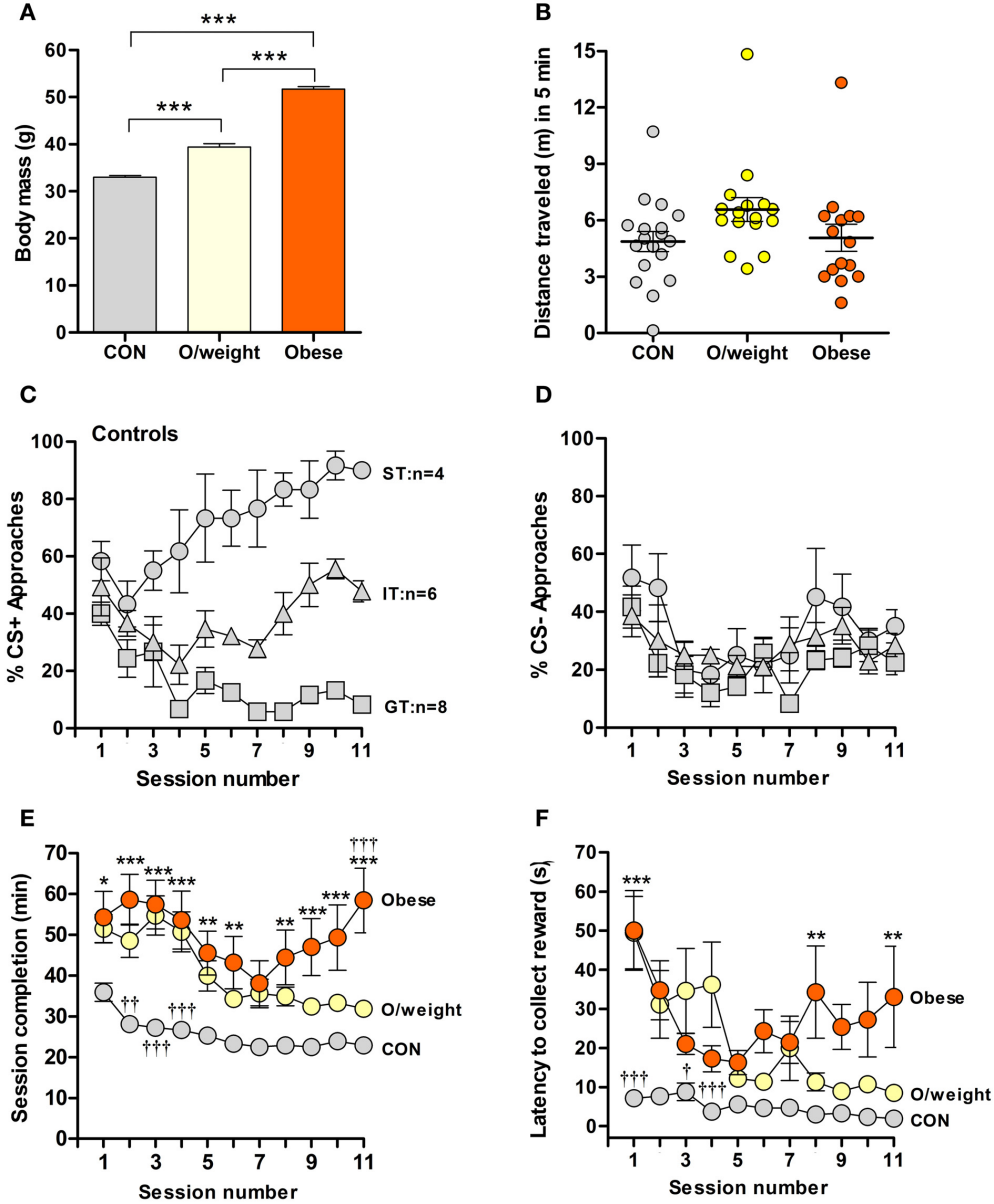
**Overweight and obese mice show poor acquisition of food-rewarded pavlovian conditioned learning. (A)** Body masses of control (CON; normal chow, *n* = 18), overweight (O/weight; low fat-high carbohydrate diet; *n* = 16) and obese mice (high fat-high carbohydrate diet, *n* = 16) at the start of experimentation. **(B)** Locomotor activity, measured in an open field arena, of CON, O/weight and Obese mice before behavioral testing commenced. **(C)** Relative number of CS+ approaches and CS- approaches **(D)** during each session; only CON mice displayed different conditioned responses (cf. Harb and Almeida, 2014), characterized as sign-tracking (ST, predominantly approached the CS+; *n* = 4), goal-tracking (GT, predominantly approached the US; *n* = 8), and intermediate-tracking (IT, alternated between CS+ and US with approximately equal frequency; *n* = 6). Autoshaping was monitored over 11 sessions; in each session, mice received 15 CS+ and 15 CS- presentations. **(E)** Time in min needed to complete successive autoshaping sessions. **(F)** Mean latency (s) to retrieve food reward during consecutive training sessions. Data are means ± s.e.m. ^***^In **(A)** denotes *p* < 0.001. ^*^, ^**^, ^***^In **(E,F)** indicate differences between CON and obese groups at *p* < 0.05, 0.01, and 0.001, respectively. ^†^, ^††^, ^†††^In **(E,F)** indicate differences between O/weight mice vs. CON and Obese mice at *p* < 0.05, 0.01, and 0.001, respectively.

To address the hypothesis that appetitive learning is altered in overweight and obese individuals, we here applied the classical pavlovian conditioning paradigm to mice that differed in body mass, reflecting their maintenance on normal chow (NC) (CON, hereinafter referred to as “control mice,” *N* = 18), low-fat/high-carbohydrate (overweight, *N* = 16) or high-fat/high-carbohydrate (obese, *N* = 16) diets. Body weights differed significantly between each of the experimental groups (*P* < 0.001, Figure 1A); none of the groups displayed motor or other behavioral impairments, as indicated by the results of testing in an open field arena (Figure 1B).

Mice were trained over 11 days (15 CS+ and 15 CS− presentations/trial session). Consistent with our earlier findings (Harb and Almeida, 2014), control mice showed conditional learning, albeit by developing three distinct types of CR: sign tracking (ST) and goal tracking (GT) where animals predominantly approach the CS+, food (US) magazine, respectively, and intermediate tracking (IT) where animals alternate between CS+ and food (US) magazine with approximately equal frequency (Figure 1C); on the other hand, the approaches toward the CS- was similar in all animals (Figure 1D). Strikingly, the overweight and obese mice did not develop the same pattern of responses as the controls; rather, the majority of them displayed IT behaviors (11/16 overweight mice and 11/16 obese mice; Supplementary Table 1), indicating that these groups made only weak associations between the light stimulus (CS+) and the food reward.

Given the above observations and to examine whether the different CR patterns of the three groups reflected reactivity to the test set-up, rather than differences in learning *per se*, we compared session completion times and latencies to reward collection. Control, overweight and obese animals differed significantly in the time taken to complete the training sessions [*F*_(2, 429)_ = 119.3; *P* < 0.0001; Figure 1E]. During all sessions, obese mice took significantly longer to complete the training session, as compared to control mice (session 1: *P* < 0.05; sessions 2–4, 9–11: *P* < 0.001; sessions 5, 6, 8: *P* < 0.01). Generally, the overweight animals were also slower than control mice in session completion (session 2: *P* < 0.01; sessions 3, 4: *P* < 0.001), and significantly faster than the obese group on the last day of training (session 11: *P* < 0.001). Between-group differences were also detected in terms of another test parameter, namely, latency to collect reward [*F*_(2, 409)_ = 52.8; *P* < 0.0001; Figure 1F]. *Post-hoc* analysis revealed shorter latencies in control *vs.* overweight mice during sessions 1 (*P* < 0.001), 3 (*P* < 0.05), and 4 (*P* < 0.001); reward collection also occurred faster in control vs. obese mice during sessions 1 (*P* < 0.001), and 8 and 11 (*P* < 0.001).

Together, the above sets of data show weaker acquisition of an appetitive learning task by overweight and obese mice, possibly due to overall reduced reactivity to the task.

### Operant conditioning performance declines with increasing body mass

In an attempt to better understand the results obtained in the pavlovian conditioning experiments, we tested the performance of control, overweight and obese mice in an operant (instrumental) conditioning paradigm. Operant conditioning is another form of associative learning which, in contrast to pavlovian conditioning, depends on reinforcement of an action (here, nose-poking the illuminated area of a touchscreen) with an outcome (here, sweetened milk); the reinforcement is strengthened over time, thus increasing the probability of action-outcome events (Balleine and Dickinson, 1998). Testing was done over 9 consecutive daily sessions, each comprised of 20 trials (20 presentations of the light stimulus). The criterion was that all 20 trials in a session should have been completed within 20 min on 3 consecutive days.

**Figure 2:**
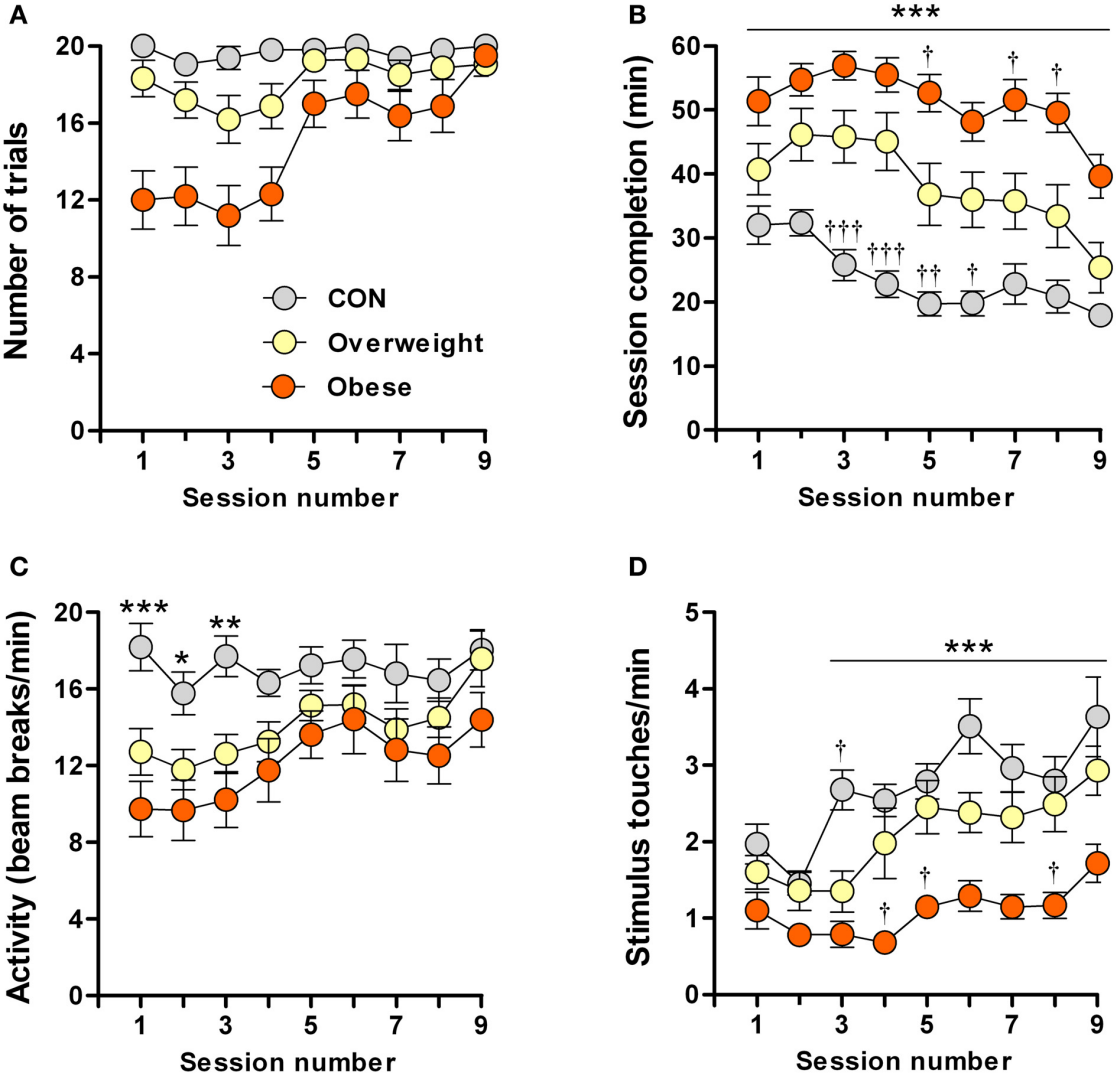
**Impaired learning of a food-rewarded operant conditioning task by overweight and obese mice**. Control (CON), overweight (O/weight) and obese mice (*n* = 16/group) were tested in 9 consecutive sessions, each consisting of 20 trials. Data shown are **(A)** Number of trials completed per test session; **(B)** Time (min) required to complete each successive session; **(C)** Locomotor activity (infra-red beam breaks/min) in touchscreen test chamber; **(D)** Number of stimulus touches/min. Data is presented as means ± s.e.m. ^*^, ^**^, ^***^Indicate significant differences between CON and obese groups (*p* < 0.05, 0.01, and 0.001, respectively). ^†^, ^††^, ^†††^Denote significant differences between overweight mice vs. CON and obese mice (*p* < 0.05, 0.01, and 0.001, respectively).

Most (87.5%, 14/16 mice) control mice reached criterion, i.e., were efficiently conditioned. However, only 43% (7/16 mice) of the overweight mice and none (0%, 0/16 mice) of the obese mice were conditioned (Supplementary Table 2). Session completion rates differed significantly between animals of different body mass in the following (increasing) rank order: controls (vs. overweight mice in sessions 3, 4: *P* < 0.001; session 5: *P* < 0.01; session 6: *P* < 0.05; and vs. obese mice in all sessions: *P* < 0.001), overweight (vs. obese mice in sessions 5, 7, 8: *P* < 0.05) and obese (Figure 2B). Notably, the obese group was slower in acquiring the task, with none of the animals in this group being able to complete all 20 trails/session during the first 4 days of testing (Figure 2A). On the other hand, despite overall (all sessions) significant between-group differences [*F*_(2, 405)_ = 150.9; *P* ≤ 0.0001], all groups took progressively less time to complete the task [*F*_(8, 405)_ = 7.4; *P* ≤ 0.0001] (Figure 2B).

To exclude impairments in motor activity and/or lack of interest that could potentially account for the slower learning by overweight and obese mice, we monitored the rate of photobeam breaks and stimulus touches (nose-pokes). While the overweight and obese mice were less mobile (fewer photobeam breaks), as compared to controls, during the first three test sessions, none of the groups differed in locomotor activity between sessions 4 and 9 (Figure 2C). Although ANOVA revealed a significant overall increase in stimulus nose-poking over time [*F*_(8, 405)_ = 9.1; *P* ≤ 0.0001]; Figure 2D shows that this increase was mainly attributable to the control and overweight groups [*F*_(2, 405)_ = 77.8; *P* ≤ 0.0001], the obese animals showing significantly fewer nose-pokes than controls during sessions 3–9 (*P* < 0.001) and overweight mice during sessions 4, 5, and 8 (*P* < 0.05).

The results from these experiments indicate that higher body mass is associated with reduced motivation in an operant task in which food is provided as the reward; our results rule out impaired motor ability in overweight and obese mice, but do not exclude the possibility that they suffer from a deficit in motivation to work for food.

### Altered motivation for food in overweight and obese mice

Here, we specifically asked whether overweight and obese animals responded differently to controls in the food conditioning experiments because of a lack of motivation toward food stimuli, i.e., if their slower acquisition of the pavlovian conditioning paradigm, in which food served as the reward, was due to their reduced interest in food *per se*. Food-restricted animals were tested on two consecutive days (1 session/day). We assessed three parameters (latencies to approach the reward and retrieve it, and number of food-tray entries) that inform on motivation for food reward (sweetened milk, 15 deliveries/session).

**Figure 3:**
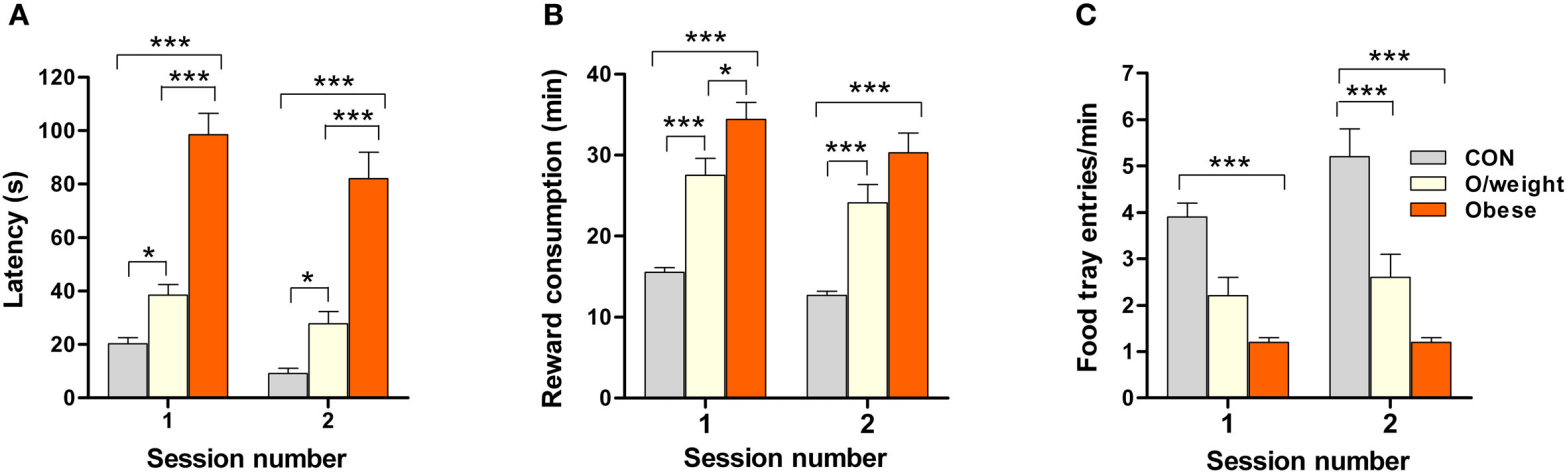
**Overweight and obese mice are less motivated to collect palatable, but low-energy, food rewards**. The sweetened milk reward was delivered 15 times in each session. The mean latencies to approach the reward **(A)**, times taken to retrieve (and consume) the reward **(B)**, and number of food tray entries **(C)** by CON (*n* = 18), overweight (O/weight, *n* = 14) and obese (*n* = 9) mice are shown (error bars represent s.e.m.). ^*^, ^***^Represent significant differences between indicated groups at *p* < 0.05 and 0.001, respectively.

Body mass had a significant impact on approach latency [*F*_(2, 71)_ = 137.5; *P* ≤ 0.0001; Figure 3A] and the time elapsed before reward retrieval [*F*_(2, 74)_ = 63.1; *P* ≤ 0.0001; Figure 3B]. The obese mice approached the reward with a significant delay, as compared to the overweight (*P* < 0.001) and control (*P* < 0.001) mice; overweight mice also showed a higher approach latency than controls (*P* < 0.05) (Figure 3A). Likewise, the rate of food reward retrieval and consumption was highest in controls > overweight > obese mice (Figure 3B). Monitoring the rate of food-tray entries as an additional index of motivation for food, revealed significant between-group differences [*F*_(2, 72)_ = 25.3; *P* ≤ 0.0001]; the highest rate was seen in controls > overweight > obese mice (Figure 3C).

The above findings suggest that lower motivation for an appetitive reinforcer, rather than impaired learning ability, can account for the poorer performance of overweight and obese mice in the pavlovian and operant learning tasks.

### Mice adjust their consumption of palatable foods according to body mass

This experiment sought to examine whether the lower motivation seen in overweight and obese mice is related to their hedonic preference for palatable foods or to their higher body mass which, in turn, implies their higher energy depots (Hariri and Thibault, 2010).

**Figure 4:**
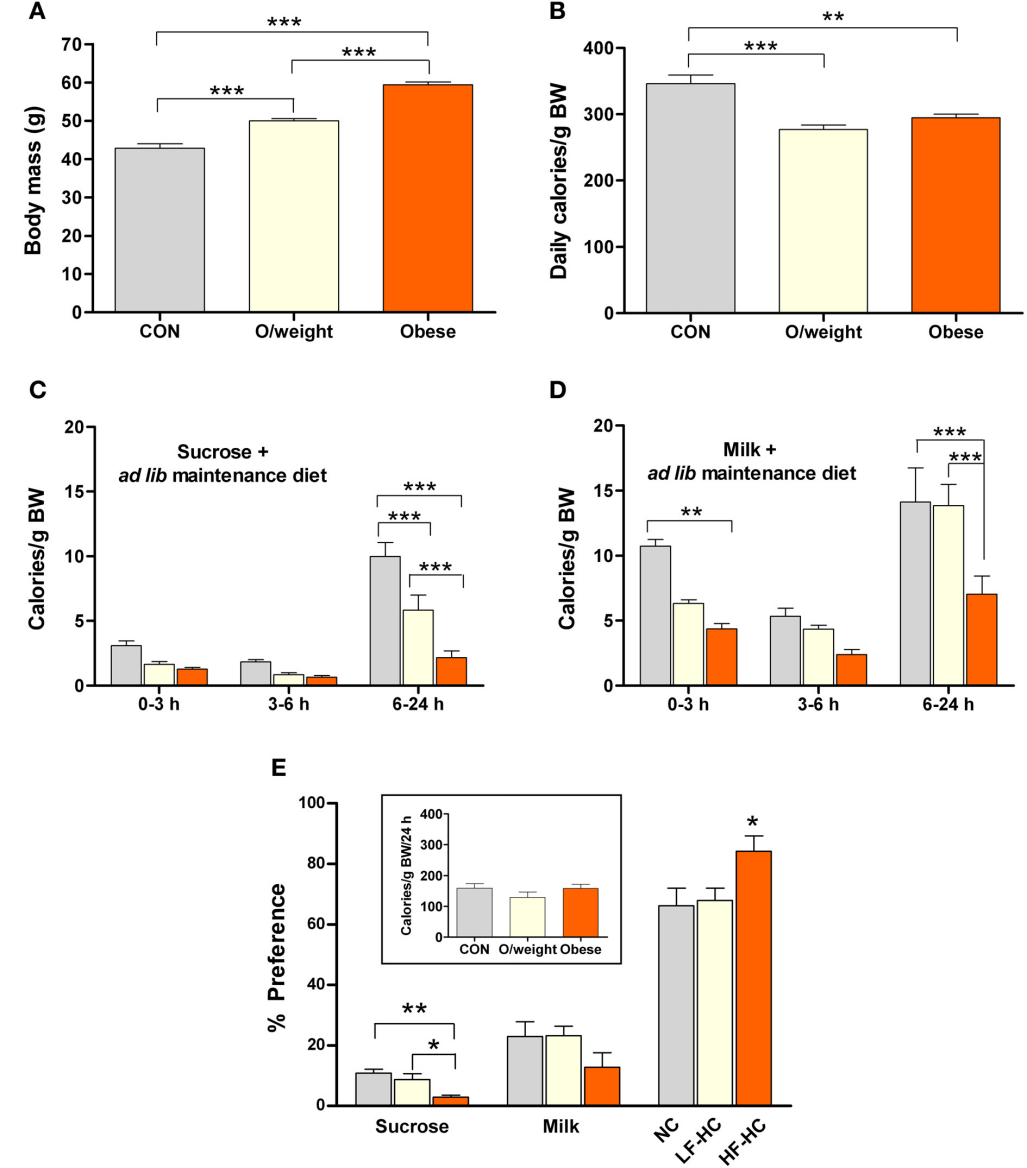
**Mice of differing body masses are sensitive to the rewarding properties of both, low-calorie foods (isocaloric 15% sucrose solution and milk containing 5% fat) and energy-dense solid chow**. Ingestion of the different foods was monitored in control (CON, *n* = 15), overweight (O/weight, *n* = 18) and obese (*n* = 14) mice during hours 0–3, 3–6, and 6–24 of presentation of the liquid foods *and* their maintenance solid diet (NC, LF-HC, HF-HC). **(A)** Body masses of the 3 groups of mice at the start of the experiment. **(B)** Average daily ingestion of calories from maintenance diets, corrected for body weight; data from 3 consecutive 24 h periods. **(C,D)** Body mass-corrected calories derived from sucrose or milk consumption over a 24 h period. **(E)** Preferences of CON, O/weight and obese mice for sucrose, milk and maintenance diet. The *inset* shows the total amount of energy ingested (maintenance diet + sucrose + milk) over 24 h. Depicted data are means ± s.e.m. ^*^, ^**^, ^***^Represent significant differences between indicated groups at *p* < 0.05, 0.01, and 0.001, respectively.

In a first step, we monitored the 24 h consumption of two isocaloric liquid foods (15% sucrose and milk with a 5% content of fat) by 12-month old control, overweight and obese mice that had *ad lib* access to the experimental (NC, HF-HC, or LF-HC) diets on which they had been maintained for 36 weeks. The three experimental groups differed in body weight (control: 42.8 ± 1.2 g; overweight: 49.9 ± 0.6 g; obese: 59.4 ± 0.8 g Figure 4A). The groups also differed in their average daily intake of calories (relative to body weight, monitored over 3 consecutive days), with the controls ingesting significantly more calories than the overweight and obese groups (*P* < 0.01; Figure 4B).

The temporal patterns of consumption of sucrose and milk by control, overweight and obese mice are depicted in Figures 4C,D. Overall, the data show that, in contrast to humans/primates and rats (Levine et al., 2003; Naleid et al., 2008), mice prefer milk over sucrose. Nevertheless, all treatment groups consumed the sucrose solution (Figure 4C), with control mice ingesting significantly more than the overweight and obese groups between 6 and 24 h (*F* = 24.7; *P* ≤ 0.0001); interestingly, the overweight mice ingested significantly more sucrose than their obese counterparts (*P* < 0.001). Obese mice consumed the least amount of milk, as compared to the control and overweight groups (6–24 h: *F* = 15.3; *P* ≤ 0.0001; obese vs. control: *P* < 0.001; obese vs. overweight: *P* < 0.001) (Figure 4D).

Expression of the total calorie intake, derived from the two liquid diets (sucrose and milk) and solid chow (NC, LF-HC, HF-HC), as a ratio of body weight (calories/g BW) revealed that control, overweight and obese mice consumed a similar relative number of calories during the 24 h test period (*inset*, Figure 4E). As shown in Figure 4E, all animals derived the majority of their daily calories from their respective solid diets >> milk > sucrose (*P* < 0.001). Notably, the relative intake of calories from solid diet was significantly higher in the obese (HF-HC) vs. control (NC) and overweight (LF-HC) (*P* < 0.05) mice, and the relative intake of calories from sucrose was significantly lower in obese vs. control (*P* < 0.01) and overweight (*P* < 0.05) mice; these findings indicate that obese mice prefer the HF-HC diet over the hedonically-loaded foods. Given the different durations of the appetitive learning and hedonic preference tests, it was considered necessary to confirm the palatability of the HF-HC foods by comparing the preferences of mice placed on this diet for 6 or 36 weeks; as shown in Supplementary Figures 1, 2, duration of exposure to HF-HC did not influence hedonic preference.

**Figure 5:**
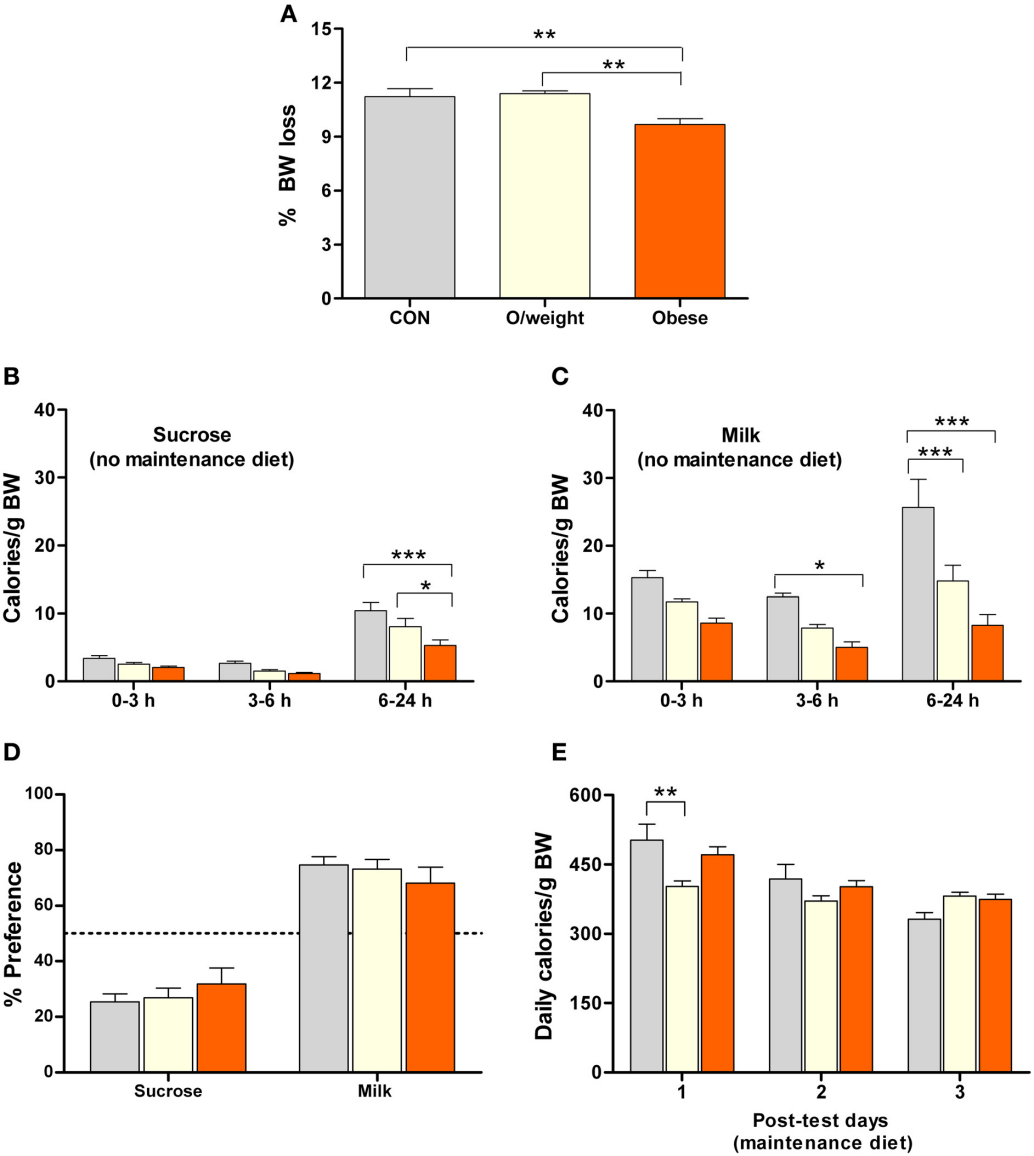
**Overweight and obese mice display differential preferences for isocaloric foods that differ in their sensory (hedonic) properties**. Isocaloric sucrose (15%) and milk (5% fat) were presented to control (CON, *n* = 15), overweight (O/weight, *n* = 18) and obese (*n* = 14) mice that had been deprived of their maintenance solid diet (NC, LF-HC, HF-HC) for 48 h. **(A)** Relative (%) body mass loss after 48 h food deprivation. **(B,C)** Calories derived from sucrose and milk over a period of 24 h. **(D)** Relative preference for sucrose and milk over 24 h [(calories derived from sucrose or calories derived from milk/total calories ingested) ^*^ 100]. **(E)** Average number of calories derived from maintenance diet food on the 3 consecutive post-test days. Means ± s.e.m. are shown. ^*^, ^**^, ^***^Denote significant (pair-wise) differences, where *p* < 0.05, 0.01, and 0.001, respectively.

The results of the above experiments suggested that food consumption in mice is based on the likelihood that the energy density of a particular food will fulfill its energy needs, rather than the sensory rewarding properties of that food. To explore this idea, we repeated the above food preference paradigm in control, overweight and obese mice that were previously food- deprived for 48 h (and did not have access to their respective solid diets during testing). This pretreatment was chosen to increase the motivation to eat as well as induce a relative energy deficit in all animals. As predicted, food deprivation caused a loss of body weight in all groups, the largest losses being observed in control and overweight mice (*P* < 0.01 vs. obese mice; Figure 5A). Again, all treatment groups consumed fewer calories from sucrose (Figure 5B) than milk (Figure 5C), confirming their preference for milk (the liquid diets were isocaloric; preference in controls > overweight > obese mice; [*F*_(2, 132)_ = 25.2; *P* < 0.0001]. The identical preference for milk over sucrose, by control, overweight and obese mice (Figure 5D) demonstrates that the latter two groups do not have a reward deficit. Lastly, all groups of animals consumed a similar number of calories on 3 consecutive days at the end of the test phase of the experiment, at which time they were returned to their respective solid diets (controls: normal chow; overweight: LF-HC; obese: HF-HC), as shown in Figure 5E.

In summary, the above findings show that increased body weight is not accompanied by a deficit in reward-responding (cf. Figures 4, 5), and that mice have the capacity to regulate their food choices in a manner that maintains constant caloric intake relative to body mass (Figure 4). The last point is reinforced by analysis and scrutiny of the data obtained in the tests of preference under conditions of *ad lib* access to one of three diets (NC, LF-HC, and HF-HC, cf. Figure 4) or under when animals were deprived of any solid food for 48 h (cf. Figure 5).

**Figure 6:**
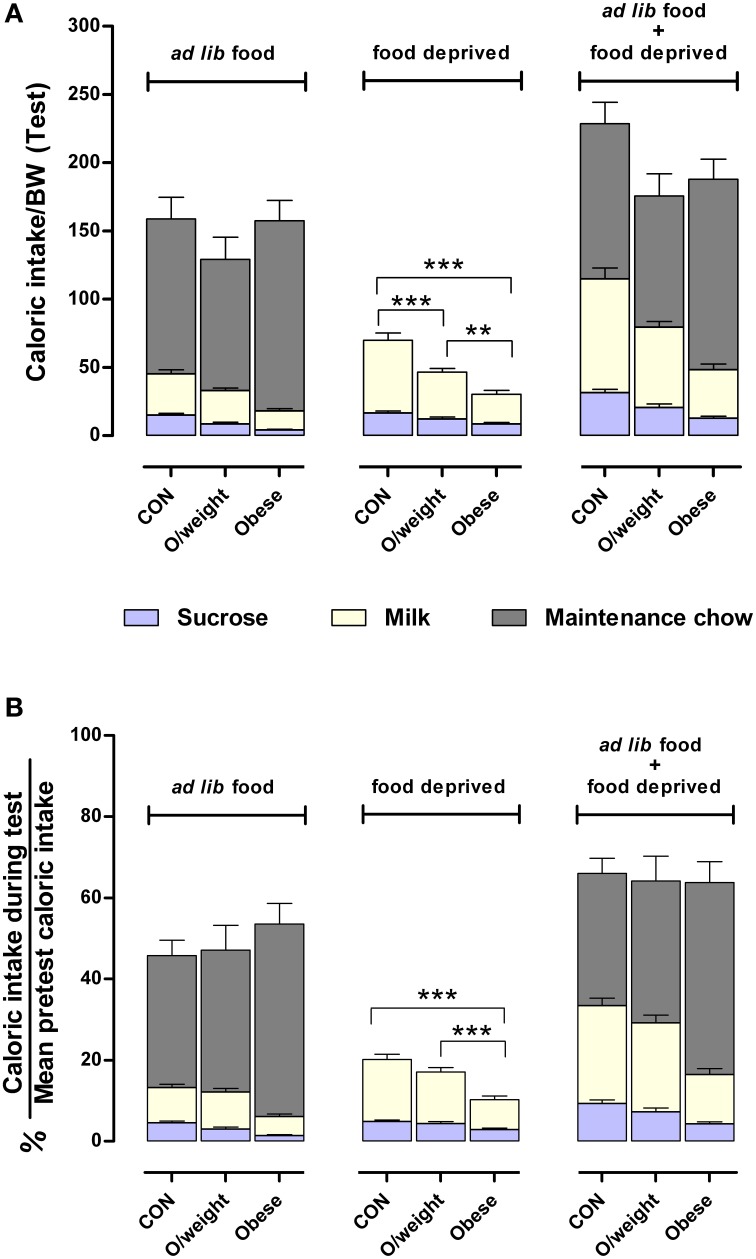
**Mice adjust their intake of different foods to maintain a similar daily caloric intake relative to body mass**. Comparisons between control (CON), overweight (O/weight) and obese mice are based on data depicted in Figures 4, 5. **(A)** Shows caloric intake during 24 h test phase from sucrose and milk, relative to body mass either in the presence of *ad lib* maintenance diet (NC, LF-HC, HF-HC) or in the absence of maintenance diet (food-deprived). **(B)** Shows caloric intake from sucrose and milk in *ad lib* presence or absence of maintenance diet, as a percentage of the average daily number calories consumed under standard feeding (solid chow only) conditions. Data shown are means ± s.e.m.; significant pair-wise differences are denoted by ^**^p < 0.01 and ^***^p < 0.001.

Lastly, we calculated the relative amount of energy intake derived from each of the respective liquid and solid diets (controls: sucrose, milk and NC; overweight: sucrose, milk and LF-HC; obese: sucrose, milk and HF-HC) with respect to each group's average daily calorie intake (data in Figure 4B). As shown in Figure 6, control, overweight and obese mice can adjust the relative amounts of each liquid and solid diet in order to maintain a relatively similar daily level of calorie ingestion, irrespective of weight status. Together, these results show that animals with higher body mass do not have a reward deficit syndrome but neglect the otherwise highly-rewarding milk and sucrose in favor of their energy-denser solid foods.

## Discussion

The behavioral mechanisms that lead to overeating and thus, overweight and obesity in humans, are still poorly understood. There are two prevailing hypotheses that are not necessarily mutually exclusive. The first posits that, overeating represents an addiction to food (compensation for an underlying reward deficit syndrome) (Wang et al., 2004; Blum et al., 2006; Stice et al., 2008; Geiger et al., 2009); the second suggests that physiological controls and signals of satiety are overridden by the hedonic (orosensory) properties of foods (Hariri and Thibault, 2010; Berridge and Kringelbach, 2011). In a previous study in mice, a species increasingly used in research to understand human obesity (Speakman et al., 2007), we presented evidence that failed to support the addiction hypothesis of overeating (Harb and Almeida, 2014). The present study addresses two key facets of the hedonic overdrive hypothesis, namely, motivation and learning. These aspects are also pertinent since global cognitive (but not executive) function is reportedly disturbed in obese humans (Gunstad et al., 2010). However, a recent meta-analysis concluded that, whereas executive function may be compromised by obesity in children and adolescents, obesity does not have clear effects on other cognitive domains, such as learning and memory (Liang et al., 2014). Notably, increased body weight and sweet or fatty (high-calorie) diets have been shown to have a negative impact on the performance of laboratory animals in some (Farr et al., 2008; Stranahan et al., 2008; Jurdak and Kanarek, 2009; Ross et al., 2009; Heyward et al., 2012; Valladolid-Acebes et al., 2013; Beilharz et al., 2014) but not all (Mielke et al., 2006; Ross et al., 2009; Hwang et al., 2010; Heyward et al., 2012; Valladolid-Acebes et al., 2013; Beilharz et al., 2014) tests of hippocampus-dependent spatial, recognition and fear learning and memory.

Our experiments show that overweight and obese mice perform poorly in pavlovian conditioning and operant conditioning, two paradigms that test appetitive learning paradigms. Complementary assessments of motivation revealed that this apparent impairment in learning ability results from the diminished motivation of overweight and obese animals to ingest food rewards. Importantly, we demonstrated that the reduced motivation to consume a food reward reflects reduced interest in appetitive reinforcers (sucrose and milk) that, although usually considered to be highly palatable and preferred (Lucas et al., 1998), contain less energy than maintenance (NC, LF-HC, HF-HC) chow in the amounts provided in the present experimental setting (cf. Figure 5: reinforcers presented in the absence of maintenance diet; Figure 4, reinforcers and maintenance diets available). A previous independent investigation, done in a different context, in obese mice concluded that weight gain can occur despite reduced motivation to retrieve a hedonic food when the cost of acquiring energy-dense foods is low (Frazier et al., 2008). Together, these findings indicate that overweight and obese mice do not suffer from a reward deficit syndrome (cf. Huang et al., 2005; Fulton et al., 2006; Davis et al., 2008; Stice et al., 2008; Geiger et al., 2009); they can sense and respond to both, the sensory and energy signals elicited by foods, but are more likely to select foods that will match their metabolic status and fulfill their energetic demands. Nevertheless, it is important to consider the results of a study by Johnson and Kenny (2010) which concluded that animals can display a reward deficit. Using a distinctly different experimental paradigm to that used in the present study, the authors reported that rats/mice that have been exposed for an extended period of time to a cafeteria diet comprised of energy-dense foods (e.g., bacon, sausage, cheesecake) show behaviors that resemble those seen in the addiction-like adaptive response to drugs of abuse.

The fact that overweight and obese animals worked less (i.e., were less motivated) for hedonically-loaded foods (sucrose, milk) may be explained by their greater energy depots stored in fat (Hariri and Thibault, 2010). This interpretation is supported by previously-observed lower motivation for an otherwise highly-palatable food in obese rats (Shin et al., 2011). Taken together, it thus appears that mice can adjust their food choices (in terms of hedonic and energetic properties) according to their actual energy needs. This point is illustrated by our observation that control, overweight and obese only differ in the amount of energy derived from individual foods, rather than in the total number of body weight-adjusted calories ingested (Figure 6). It is important to note here that, laboratory animals may differ from humans in that they are less exposed to environments where hedonic signals abound and can override actual metabolic demands.

The present findings raise important questions regarding the interpretation of results from overweight and obese rodents in which learning and memory is assessed using paradigms in which food is used as the reinforcing stimulus (e.g., Greenwood and Winocur, 1990; McNeilly et al., 2001; Mielke et al., 2006; Farr et al., 2008; Murray et al., 2009; Valladolid-Acebes et al., 2011). The apparent impaired ability of overweight and obese animals in such tests may simply reflect their reduced motivation (reduced “wanting”) to retrieve and consume appetitive rewards, illustrated by our results from a motivation task that did not depend on learning ability (Figure 3). This interpretation relates to behavior in animals that are *already* obese or overweight, and not to the initiation of these states which result from multifactorial physiological and behavioral mechanisms (see Hariri and Thibault, 2010).

In summary, our experiments indicate that appetitive learning mechanisms are intact in overweight and obese animals, although over-shadowed by alterations in motivation (not reward insensitivity or reward deficit) for foods that may be hedonically less-attractive but more likely to meet the organism's metabolic needs. Unlike humans, mice eat according to their metabolic need rather than simply respond to the hedonic properties of food. Our findings also show that extrapolation of results from studies reporting learning deficits in overweight/obese rodents to humans require caution; whereas most tests of learning ability in rodents employ appetitive stimuli, learning deficits in humans are detected using tests that are not confounded by the use of food-related stimuli. Lastly, translational studies need to recognize that humans are more exposed to reinforcing conditioning stimuli than laboratory animals and are therefore more likely to lose control over eating and gain excess weight.

## Author contributions

Mazen R. Harb and Osborne F. X. Almeida designed the study. Mazen R. Harb performed the experiments and collected and analyzed the data. Mazen R. Harb and Osborne F. X. Almeida wrote the paper. Both authors read and approved the final manuscript.

### Conflict of interest statement

The authors declare that the research was conducted in the absence of any commercial or financial relationships that could be construed as a potential conflict of interest.
