# Supplementary material for: Altered motivation masks appetitive learning potential of obese mice
Source: Front Behav Neurosci. 2014 Oct 30;8:377. doi: 10.3389/fnbeh.2014.00377 (PMC4214228; doi:10.3389/fnbeh.2014.00377)
Supplement: Supplementary file 1 [file Table1.PDF]

Supplementary Table 1: Distribution of conditioned responses (CR) in control, overweight and obese mice. Mice of normal body weight (control, maintained on standard lab chow, n = 18) developed 3 distinct types of conditioned responses (Sign Tracking [ST], Goal Tracking [GT] or Intermediate Tracking [IT]). The majority of overweight (maintained on a low fat-high carbohydrate [LF-HC] diet, n = 16) and obese (maintained on a high fat-high carbohydrate [HF-HC] diet, n =16) mice displayed IT behavior.

Conditioned response (CR)	Control	Overweight	Obese
Sign tracking	4	2	0
Goal tracking	8	2	0
Intermediate	6	11	11
Task failed	0	1	5
