# Supplementary material for: Altered motivation masks appetitive learning potential of obese mice
Source: Front Behav Neurosci. 2014 Oct 30;8:377. doi: 10.3389/fnbeh.2014.00377 (PMC4214228; doi:10.3389/fnbeh.2014.00377)
Supplement: Supplementary file 2 [file Table2.PDF]

Supplementary Table 2: Relative numbers of animals that reached the criterion in the operant conditioning paradigm. Three groups of mice differing in body weight were tested: Control (maintained on standard lab chow, n = 16), overweight (maintained on a low fat-high carbohydrate [LF-HC] diet, n = 16) and obese (maintained on a high fat-high carbohydrate [HF-HC] diet, n =16) were tested.

Operant conditioning	Control	Overweight	Obese
No of animals reaching criterion: completed session within 20 min on at least 3 consecutive days	87.5 % (14/16 mice)	43 % (7/16 mice)	0 % (0/16 mice)
