# Supplementary material for: Altered motivation masks appetitive learning potential of obese mice
Source: Front Behav Neurosci. 2014 Oct 30;8:377. doi: 10.3389/fnbeh.2014.00377 (PMC4214228; doi:10.3389/fnbeh.2014.00377)
Supplement: Supplementary file 3 [file Presentation1.PDF]

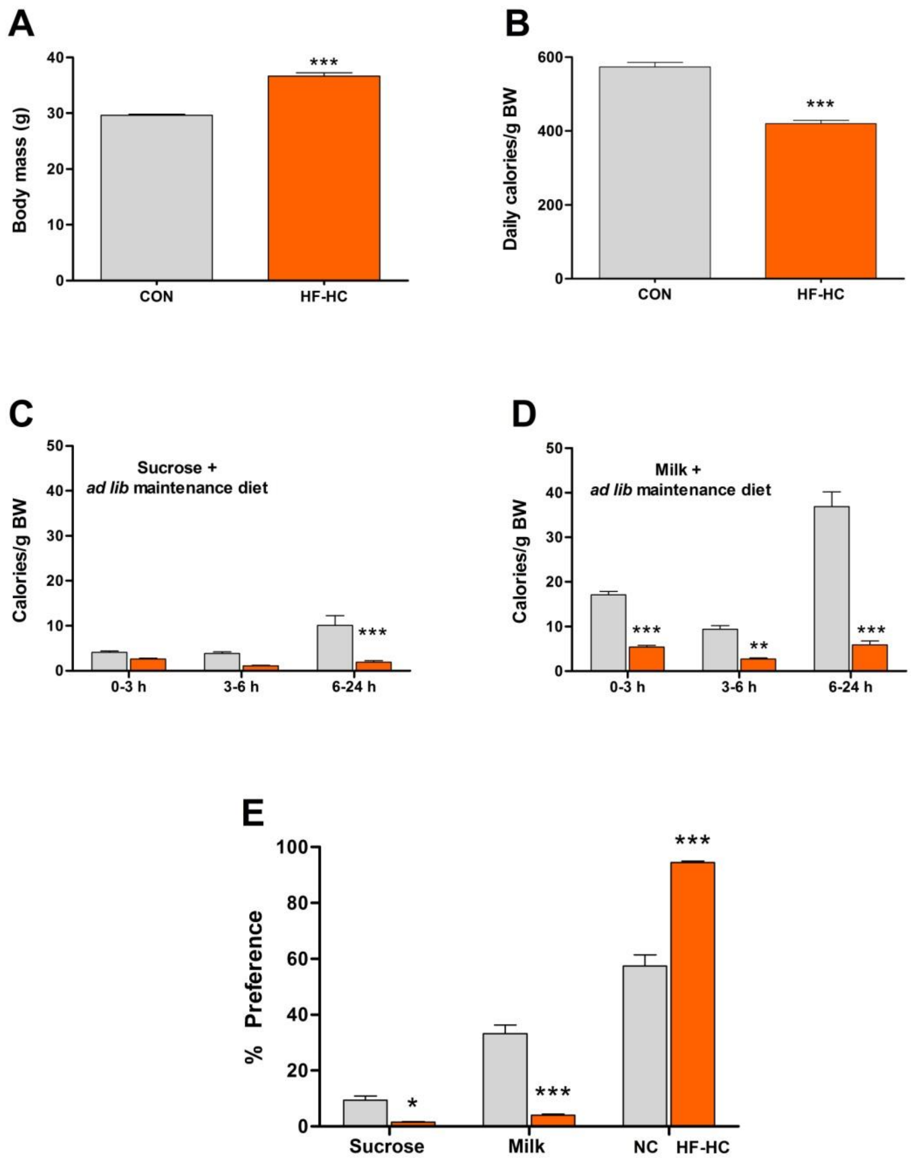

Supplementary Figure 1. Hedonic preference test in mice exposed to a high-fat, high-carbohydrate diet "HF-HC" for 6 weeks. Ingestion of the different foods was monitored in control (CON, n = 20) and HF-HC (n = 20) groups between 0-3, 3-6 and 6-24 h of simultaneous presentation of isocaloric liquid foods (sucrose and milk) *and* their respective maintenance solid diets (NC, HF-HC). **(A)** Body masses of mice at the start of the experiment. **(B)** Average daily ingestion of calories from maintenance diets, corrected for body weight over 3 consecutive 24 h periods. **(C, D)** Body mass-corrected calories derived from sucrose or milk consumption over a 24 h period. **(E)** Preferences displayed by mice in CON and HF-HC mice for sucrose, milk and maintenance diet over 24 h. Depicted data are means ± SEM. *, ** and *** represent significant differences between indicated groups at $p < 0.05$, 0,01 and 0.001, respectively.
