# Supplementary material for: Altered motivation masks appetitive learning potential of obese mice
Source: Front Behav Neurosci. 2014 Oct 30;8:377. doi: 10.3389/fnbeh.2014.00377 (PMC4214228; doi:10.3389/fnbeh.2014.00377)
Supplement: Supplementary file 4 [file Presentation2.PDF]

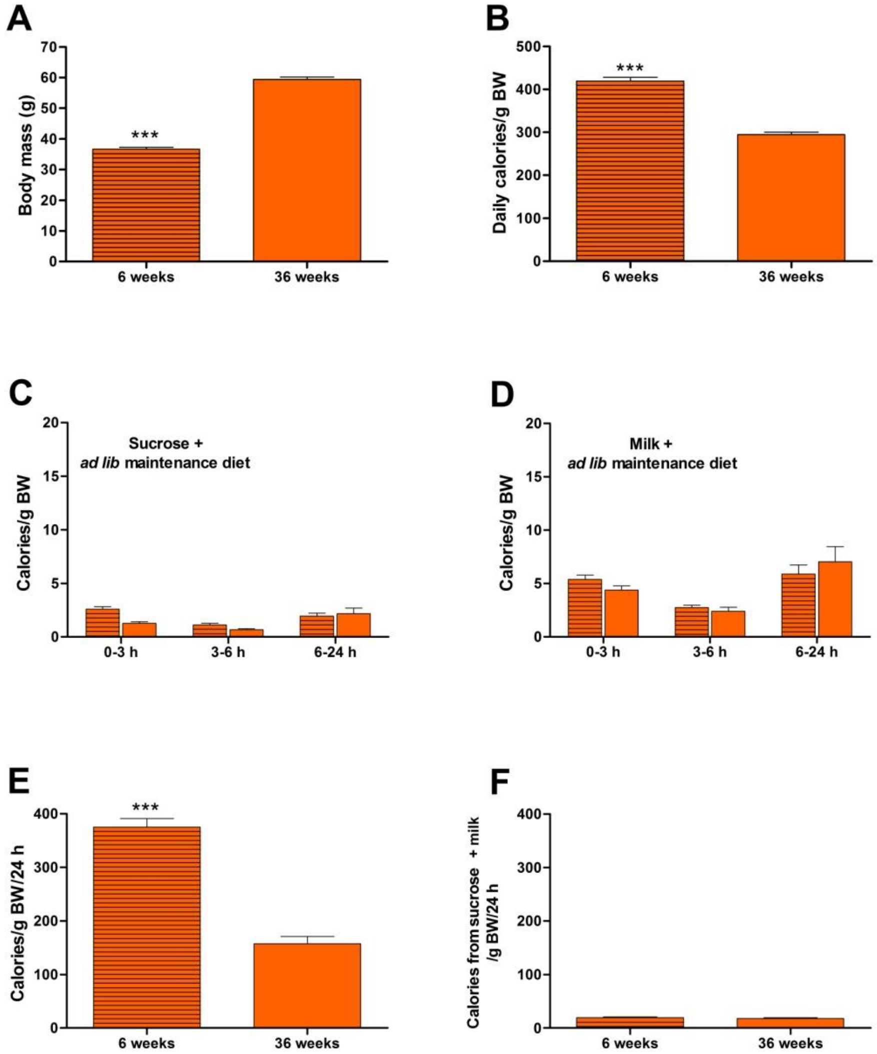

Supplementary Figure 2. Hedonic preference test in mice exposed for 6 or 36 weeks to a high-fat, high-carbohydrate diet "HF-HC". Ingestion of the different foods was monitored in mice exposed to HF-HC for 6 weeks (n = 20) and to HF-HC for 36 weeks (n = 14) at the following intervals: 0-3, 3-6 and 6-24 h of presentation of the isocaloric liquid foods (sucrose and milk) *and* the respective maintenance solid diet (HF-HC). **(A)** Body masses at the start of the experiment. **(B)** Average daily ingestion of calories from maintenance diet, corrected for body weight over 3 consecutive 24 h periods. **(C, D)** Body mass-corrected calories derived from sucrose or milk consumption over a 24 h period. **(E)** Total amount of energy ingested (maintenance diet + sucrose + milk) over 24 h. **(F)** Total amount of energy ingested from the liquid solutions (sucrose + milk) only over 24 h. Depicted data are means ± SEM. *** represent significant differences between indicated groups at $p < 0.001$.
